# 752. Costs Attributable to *Clostridioides difficile* Infection in the Presence of Differential Mortality

**DOI:** 10.1093/ofid/ofab466.949

**Published:** 2021-12-04

**Authors:** John Sahrmann, Dustin Stwalley, Margaret A Olsen, Holly Yu, Erik R Dubberke

**Affiliations:** 1 Washington University, St. Louis, Missouri; 2 Washington University in St. Louis, St. Louis, MO; 3 Pfizer, Inc, Collegeville, Pennsylvania

## Abstract

**Background:**

CDI imposes a major burden on the U.S. healthcare system. Obtaining accurate estimates of economic costs is critical to determining the cost-effectiveness of preventive measures. This task is complicated by differences in epidemiology, mortality, and baseline health status of infected and uninfected individuals, and by the statistical properties of costs data (e.g., right-skewed, excess of zeros costs).

**Methods:**

Incident CDI cases were identified from Medicare 5% fee-for-service data between 2011 and 2017 and classified into standard surveillance definitions: hospital-onset (HO); other healthcare facility-onset (OHFO); community-onset, healthcare-associated (CO-HCFA); or community-associated (CA). Cases were frequency matched 1:4 to uninfected controls based on age, sex, and year of CDI. Controls were assigned to surveillance definitions based on location at index dates. Medicare allowed costs were summed in 30-day intervals up to 3 years following index. One- and 3-year cumulative costs attributable to CDI were computed using a 3-part estimator consisting of a parametric survival model and a pair of 2-part models predicting costs separately in intervals where death did and did not occur, adjusting for underlying acute and chronic conditions.

**Results:**

60,492 CDI cases (Figure 1) were matched to 241,968 controls. Three-year mortality was higher among CDI cases compared to matched controls for HO (45% vs 26%) and OHFO (42% vs 36%), whereas mortality was slightly lower for CDI cases compared to controls for those with community onset (CO-HCFA: 28% vs 32%; CA: 10% vs 11%). One- and 3-year attributable costs due to CDI are shown in Figure 2. Adjusted 1-year attributable costs amounted to &26,954 (95% CI: &26,154–&27,939) for HO; &10,539 (&9,564–&11,518) for OHFO; &6,525 (&5,012–&8,171) for CO-HCFA; and &3,171 (&1,841–&4,200) for CA. Adjusted 3-year attributable costs were &44,736 (&43,063–&46,483) for HO; &13,994 (&12,529–&15,975) for OHFO; &7,349 (&4,738–&10,246) for CO-HCFA; and &2,377 (&166–&4,722) for CA.

Figure 1. Proportion of Cases by CDI Surveillance Definitions

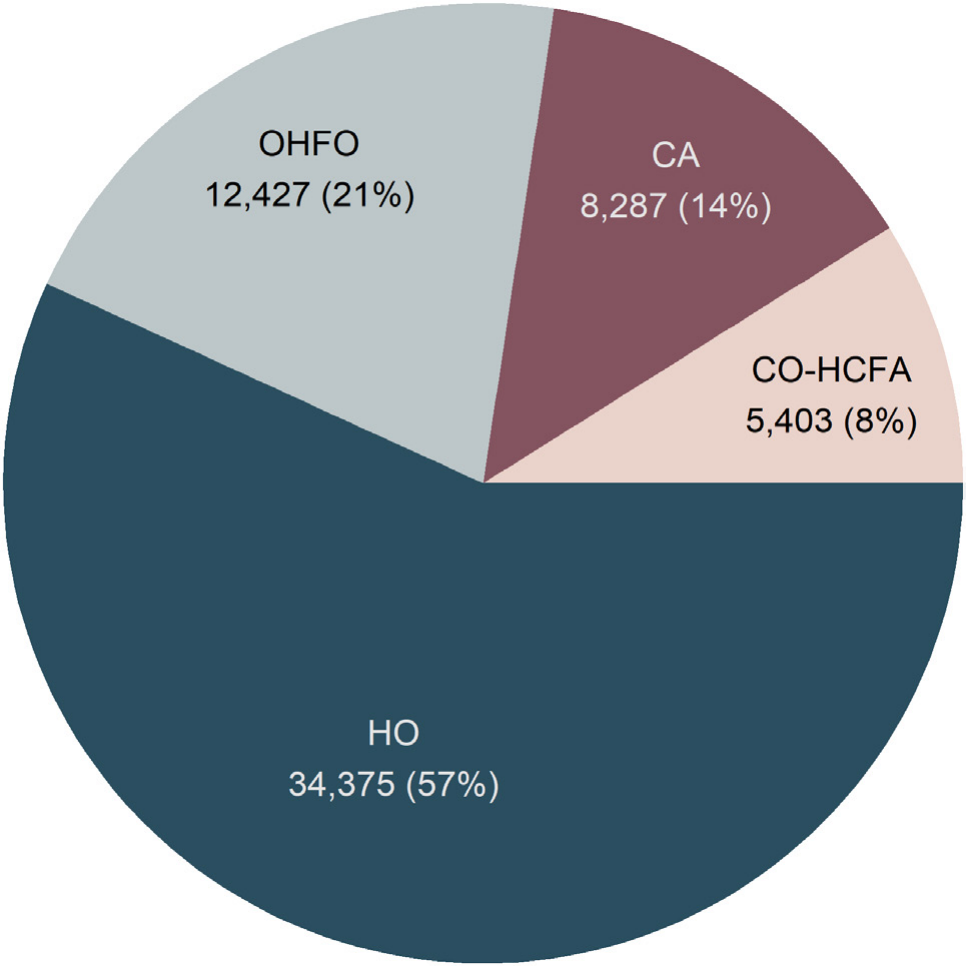

Abbreviations: HO: hospital-onset; OHFO: other healthcare facility-onset; CO-HCFA: community-onset, healthcare-associated; CA: community-associated.

Figure 2. Estimates of Costs Attributable to CDI by CDI Surveillance Definitions at One and Three Years after Onset

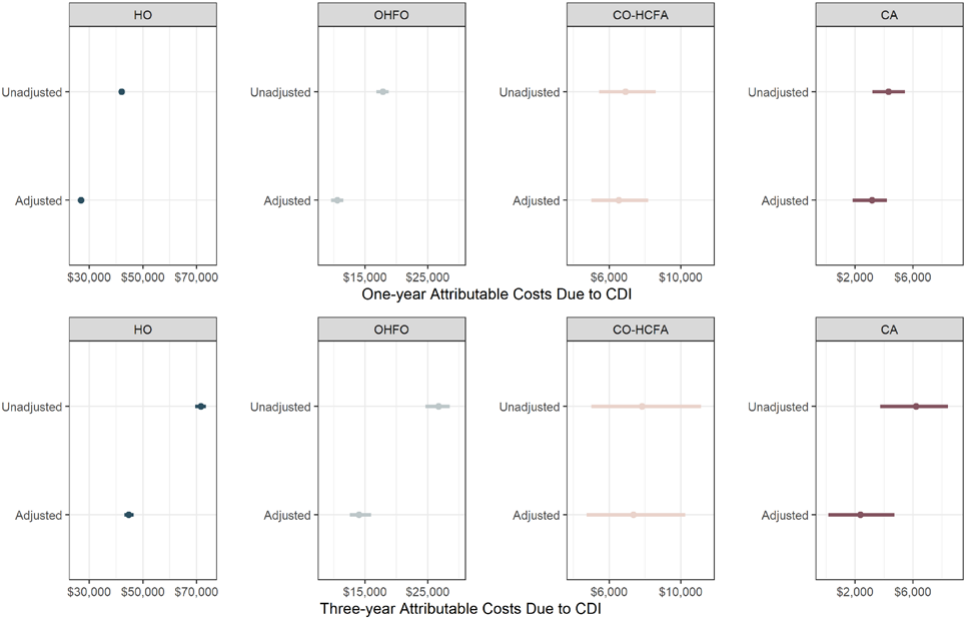

Top panels: One-year cost estimates. Bottom panels: Three-year cost estimates. Abbreviations: HO: hospital-onset; OHFO: other healthcare facility-onset; CO-HCFA:community-onset, healthcare-associated; CA:community-associated.

**Conclusion:**

CDI was associated with increased healthcare costs across surveillance definitions in Medicare fee-for-service patients after adjusting for survival and underlying conditions.

**Disclosures:**

**Dustin Stwalley, MA**, **AbbVie Inc** (Shareholder)**Bristol-Myers Squibb** (Shareholder) **Margaret A. Olsen, PhD, MPH**, **Pfizer** (Consultant, Research Grant or Support) **Holly Yu, MSPH**, **Pfizer** (Employee) **Erik R. Dubberke, MD, MSPH**, **Ferring** (Grant/Research Support)**Merck** (Consultant)**Pfizer** (Consultant, Grant/Research Support)**Seres** (Consultant)**Summit** (Consultant)

